# International Cooperation to Enable the Diagnosis of All Rare Genetic Diseases

**DOI:** 10.1016/j.ajhg.2017.04.003

**Published:** 2017-05-04

**Authors:** Kym M. Boycott, Ana Rath, Jessica X. Chong, Taila Hartley, Fowzan S. Alkuraya, Gareth Baynam, Anthony J. Brookes, Michael Brudno, Angel Carracedo, Johan T. den Dunnen, Stephanie O.M. Dyke, Xavier Estivill, Jack Goldblatt, Catherine Gonthier, Stephen C. Groft, Ivo Gut, Ada Hamosh, Philip Hieter, Sophie Höhn, Matthew E. Hurles, Petra Kaufmann, Bartha M. Knoppers, Jeffrey P. Krischer, Milan Macek, Gert Matthijs, Annie Olry, Samantha Parker, Justin Paschall, Anthony A. Philippakis, Heidi L. Rehm, Peter N. Robinson, Pak-Chung Sham, Rumen Stefanov, Domenica Taruscio, Divya Unni, Megan R. Vanstone, Feng Zhang, Han Brunner, Michael J. Bamshad, Hanns Lochmüller

**Affiliations:** 1 Children’s Hospital of Eastern Ontario Research Institute, University of Ottawa, Ottawa, ON K1H 8L1, Canada; 2 Orphanet, Institut National de la Santé et de la Recherche Médicale US14, 75014 Paris, France; 3 Department of Pediatrics, University of Washington, Seattle, WA 98195, USA; 4 Department of Genetics, King Faisal Research Center, Riyadh 11211, Saudi Arabia; 5 Saudi Human Genome Program, King Abdulaziz City for Science and Technology, Riyadh 11442, Saudi Arabia; 6 Genetic Services of Western Australia, Perth, WA 6008, Australia; 7 Department of Genetics, University of Leicester, Leicester LE1 7RH, UK; 8 Department of Computer Science, University of Toronto, Toronto M5S 1A1, Canada; 9 Genomic Medicine Group, Galician Foundation of Genomic Medicine and University of Santiago de Compostela, 15782 Santiago de Compostela, Spain; 10 Departments of Human Genetics and Clinical Genetics, Leiden University Medical Center, Albinusdreef 2, 2333 ZA Leiden, the Netherlands; 11 Centre of Genomics and Policy, Department of Human Genetics, Faculty of Medicine, McGill University, Montreal, QC H3A 1A4, Canada; 12 Experimental Division, Sidra Medical and Research Center, PO Box 26999, Doha, Qatar; 13 Genetics Unit, Dexeus Woman’s Health, 08028 Barcelona, Spain; 14 National Center for Advancing Translational Sciences, National Institutes of Health, Bethesda, MD 20892-4874, USA; 15 Centre Nacional d’Anàlisi Genòmica, Center for Genomic Regulation, Barcelona Institute of Science and Technology, Universitat Pompeu Fabra, 08028 Barcelona, Spain; 16 McKusick-Nathans Institute of Genetic Medicine, Johns Hopkins University School of Medicine, Baltimore, MD 21286, USA; 17 Michael Smith Laboratories, Department of Medical Genetics, University of British Columbia, Vancouver, BC V6T 1Z4, Canada; 18 Wellcome Trust Sanger Institute, Wellcome Trust Genome Campus, Hinxton CB10 1SA, UK; 19 Office of Rare Diseases Research, National Center for Advancing Translational Sciences, National Institutes of Health, Bethesda, MD 20892-4874, USA; 20 University of South Florida Health Informatics Institute, Tampa, FL 33620, USA; 21 Department of Biology and Medical Genetics, Second Faculty of Medicine, Charles University and University Hospital Motol, 150 06 Prague 5, Czech Republic; 22 Center for Human Genetics, University of Leuven, 3000 Leuven, Belgium; 23 Lysogene, 92 200 Neuilly-sur-Seine, France; 24 Broad Institute of MIT and Harvard, Cambridge, MA 02142, USA; 25 Institut für Medizinische Genetik und Humangenetik, Charité Universitätsmdizin Berlin, 13353 Berlin, Germany; 26 Jackson Laboratory for Genomic Medicine, Farmington, CT 06032, USA; 27 Centre for Genomic Sciences, University of Hong Kong, Hong Kong, China; 28 Department of Social Medicine and Public Health, Faculty of Public Health, Medical University of Plovdiv, Plovdiv 4002, Bulgaria; 29 National Centre for Rare Diseases, Istituto Superiore di Sanità, Rome 299-00161, Italy; 30 WuXi AppTec, Waigaoqiao Free Trade Zone, Shanghai 200131, China; 31 WuXi NextCODE, Cambridge, MA 02142, USA; 32 Department of Human Genetics, Radboud University Medical Center, 6525 GA Nijmegen, the Netherlands; 33 Maastricht University Medical Center, Department of Clinical Genetics, 6229 GT Maastricht, the Netherlands; 34 Division of Genetic Medicine, Seattle Children’s Hospital, Seattle, WA 98105, USA; 35 John Walton Muscular Dystrophy Research Centre, MRC Centre for Neuromuscular Diseases, Institute of Genetic Medicine, Newcastle University, Newcastle upon Tyne NE1 3BZ, UK

**Keywords:** IRDiRC, rare diseases, gene discovery, solving the unsolved, ontologies, Matchmaker Exchange, genome sequencing, transcriptome sequencing, disease modeling

## Abstract

Provision of a molecularly confirmed diagnosis in a timely manner for children and adults with rare genetic diseases shortens their “diagnostic odyssey,” improves disease management, and fosters genetic counseling with respect to recurrence risks while assuring reproductive choices. In a general clinical genetics setting, the current diagnostic rate is approximately 50%, but for those who do not receive a molecular diagnosis after the initial genetics evaluation, that rate is much lower. Diagnostic success for these more challenging affected individuals depends to a large extent on progress in the discovery of genes associated with, and mechanisms underlying, rare diseases. Thus, continued research is required for moving toward a more complete catalog of disease-related genes and variants. The International Rare Diseases Research Consortium (IRDiRC) was established in 2011 to bring together researchers and organizations invested in rare disease research to develop a means of achieving molecular diagnosis for all rare diseases. Here, we review the current and future bottlenecks to gene discovery and suggest strategies for enabling progress in this regard. Each successful discovery will define potential diagnostic, preventive, and therapeutic opportunities for the corresponding rare disease, enabling precision medicine for this patient population.

## Main Text

### Introduction

Rare diseases, though individually rare, are collectively common. A rare disease is defined as one that affects fewer than 200,000 people in the US^1^ or less than 1 in 2,000 people in Europe.^2^A substantive number of rare diseases are due to altered functions of single genes. Cumulatively, these rare genetic diseases (RGDs), also termed Mendelian or monogenic diseases, affect at least 1 in 50 individuals in the European-derived general population.^3^ Our understanding of the number of RGDs that exist is incomplete but is estimated to be well over 7,000 according to current medical and genetic evidence^4^ (also see Orphanet in the Web Resources). Despite their often chronic and progressive nature, long-term complications can be lessened or delayed for some RGDs if they are diagnosed early (e.g., via newborn screening) and optimally managed by standard and/or targeted therapies. In addition, a definitive molecular diagnosis can obviate the need for further diagnostic investigations, facilitate appropriate access to healthcare resources, reduce prognostic uncertainty, provide accurate recurrence-risk counseling, foster reproductive choices in affected families, and impart psychosocial benefits to the patient and their family. Importantly, understanding the underlying genetic etiology and linking a RGD to a causative biological pathway is leading to highly effective targeted therapies for some severe, previously only symptomatically treatable RGDs (e.g., ivacaftor for class III *CFTR* [MIM: 602421] pathogenic variants).^5^ Ultimately, successful deployment of precision medicine will be directly related to diagnostic success for patients with RGDs.

### Current Understanding of Phenotypic and Genetic Diversity of RGDs

Knowledge of the phenotypic and genetic diversity of RGDs is steadily increasing; however, substantial gaps remain. Establishing the number of RGDs is challenging for several reasons, not the least of which is distinguishing between novel and known diseases to objectively segment a continuum of pathologies into discrete disease entities. Two international databases curate clinical and genetic data for the community: Online Mendelian Inheritance in Man (OMIM)^4^ and Orphanet.^6^ OMIM has continuously provided curation and classification of Mendelian disease since it began as *Mendelian Inheritance in Man*, first published by Dr. V. McKusick in 1966; OMIM has been online and searchable since 1987. OMIM mines the biomedical literature and, according to expert review, curates significant new information on genes and genetic phenotypes into separate gene and phenotype entries. OMIM numbers for Mendelian diseases are incorporated into the biomedical literature across many disciplines of medicine. OMIM emphasizes gene-phenotype relationships by cataloging the same or similar phenotypes caused by pathogenic variants in different genes as distinct entities; genetic heterogeneity is displayed through the associated Phenotypic Series. A recent analysis of OMIM (data downloaded September 5, 2016) recognized 3,209 unique genes associated with 4,550 monogenic rare diseases.

Orphanet (see Web Resources) has maintained an inventory of both genetic and other rare diseases since 1997. Within Orphanet, a rare disease is defined as a recognizable and homogeneous clinical presentation, whatever the cause or the number of genes related to it. Disorders are organized in a multi-hierarchical classification and can be further subdivided into subtypes, of which genetic subtypes are included. Orphanet performs a literature survey and curates the published literature of newly discovered genes or new gene-disease relations. As a result, a semantic relation is assigned to couple the gene and disease in the database. As of September 14, 2016, Orphanet documented 3,654 unique genes associated with 3,551 rare diseases.

The discrepancy in the number of rare diseases with monogenic etiology documented in each of the two databases (4,550 for OMIM and 3,551 for Orphanet) can be attributed to the way each database is structured; OMIM categorizes rare diseases on the basis of genetic etiology, whereas Orphanet groups by clinically recognizable diseases and can include more than one OMIM entry when the same disease is caused by variants in more than one gene. Recently, the Clinical Genome Resource (ClinGen)^7^ has begun defining the strength of evidence for published gene-disease associations. The evidence levels are scored according to semiquantitative frameworks, and the scores are posted on ClinGen’s website along with the scoring sheets that structure the evidence and sources. These scores will also soon be posted on OMIM. As ClinGen grows, it will enable a clear delineation between those genes for which gene-disease causality is substantiated and those claims that will require further evidence for implication.

**Figure 1:**
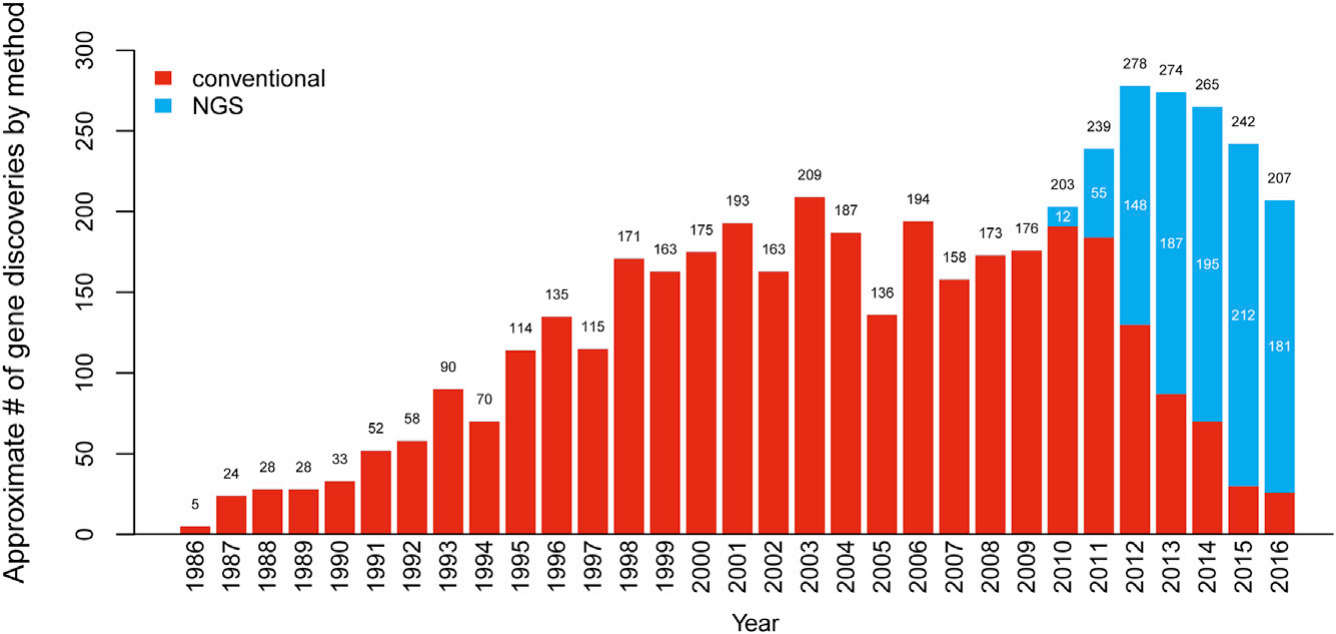
Approximate Number of Gene Discoveries Made by WES and WGS versus Conventional Approaches since 2010 according to OMIM Data Since the introduction of WES and WGS in 2010, the pace of the discovery of genes underlying RGDs per year has increased, and the proportion of discoveries made by WES or WGS (blue) or by conventional approaches (red) has steadily increased. Since 2013, WES and WGS have discovered nearly three times as many genes as conventional approaches, but the rate of discovery appears to be declining. Adapted from Chong et al.^8^

Although substantial progress has been made toward identifying the genetic basis of rare diseases, the underlying etiologies for approximately half remain undiscovered. Beginning in the mid-1980s, and for the following two decades, the primary approach to gene discovery was a combination of linkage analysis, positional cloning, and sequencing of candidate or regionally selected genes, most of which was hypothesis driven. The subsequent introduction of next-generation sequencing (NGS) strategies to identify genes associated with disease, primarily based on whole-exome sequencing (WES), in 2009 accelerated the pace of discovery by enabling hypothesis-free approaches. Today, WES is routinely used as the primary technological approach to discovering disease-gene associations (Figure 1). Its favor over whole-genome sequencing (WGS) has primarily been due to its significantly lower cost and that the majority of pathogenic variants continue to be within the protein-coding portion of the genome. Without a doubt, as the cost of WGS decreases, clinicians and researchers will transition to its use given its more even coverage, its ability to identify structural variation, and the opportunity it provides to uncover non-exomic variants.

**Figure 2:**
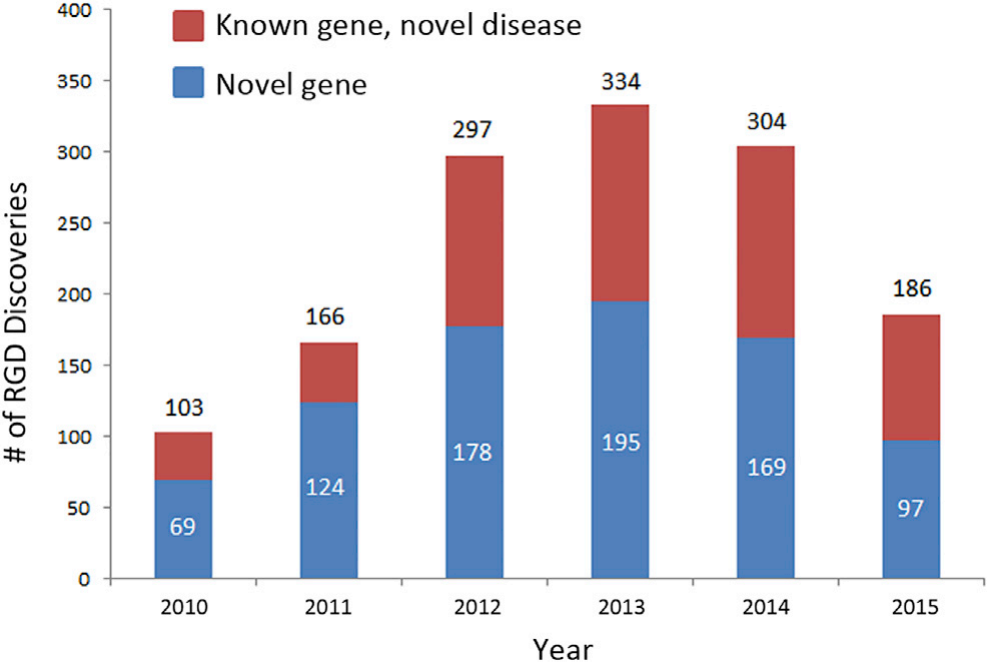
Approximate Number of Novel Gene-Phenotype Discoveries from 2010 to 2015 according to Ophanet Data Since 2010, the proportion of discoveries that are new disease-gene relations each year (known genes associated with a new disease) has steadily increased. Since 2013, the rate of discovery of both novel genes and new disease-gene relations appears to be declining.

Our analysis of OMIM documented an average of 259 “novel” RGD discoveries per year from 2012 to 2015 (Figure 1), comprising 157 new disease-gene discoveries (here defined as pathogenic variants in a gene that had not been previously associated with disease) and 102 new disease-gene relations each year (defined as pathogenic variants in a gene previously associated with a different disease; data not shown).^8^ Orphanet documents an average of 281 novel RGD discoveries per year over the same time period: 160 new disease-gene discoveries and 121 new disease-gene relations (Figure 2). Orphanet and OMIM report essentially the same number of new disease-gene discoveries (average of 160 and 157, respectively, over the same time period), but more disease-gene relations have been reported by Orphanet (121 versus 102 for OMIM). In a manual review of randomly selected discrepancies between OMIM and Orphanet, this is most likely attributable to differences in the process of curation; OMIM is more likely to decide that the publication reports a phenotypic expansion of an already explained RGD than a new disease-gene relation. Nevertheless, the data from OMIM and Orphanet both show that a significant proportion of RGD discoveries are new diseases associated with pathogenic variants in previously known genes (gene-disease relations): 38 and 43%, respectively. This is an interesting trend in comparison with a recent analysis of all of OMIM’s data, which demonstrated that nearly 25% of all genes associated with Mendelian disease underlie two or more clinically distinct disorders.^8^

Since the introduction of WES, many RGDs that were previously intractable to conventional gene-discovery approaches, largely because they were associated with a substantially reduced reproductive fitness, have been found to be caused by de novo pathogenic variants or to exhibit high allelic or locus heterogeneity. These RGDs are enriched with highly recognizable clinical presentations; are often associated with early age of onset, severe phenotype, and/or clear laboratory and/or medical imaging features; and are caused by highly penetrant pathogenic, protein-coding genomic variants (i.e., in legacy terminology, “mutations”). In addition, these RGDs are usually autosomal, X-linked recessive, or de novo dominant, rendering them relatively more accessible and amenable to current discovery strategies relying on WES; these RGDs represent the sweet spot of WES-based approaches. Both OMIM and Orphanet data (Figures 1 and 2) show a trend toward a decreasing number of discoveries per year; whether this trend is real or will continue will require analysis of data from future years. However, what is clear is that recognized bottlenecks must be addressed if the current pace of discoveries is to be maintained, or even accelerated, after the more straightforward RGDs have been solved.

### The International Rare Diseases Research Consortium

**Figure 3:**
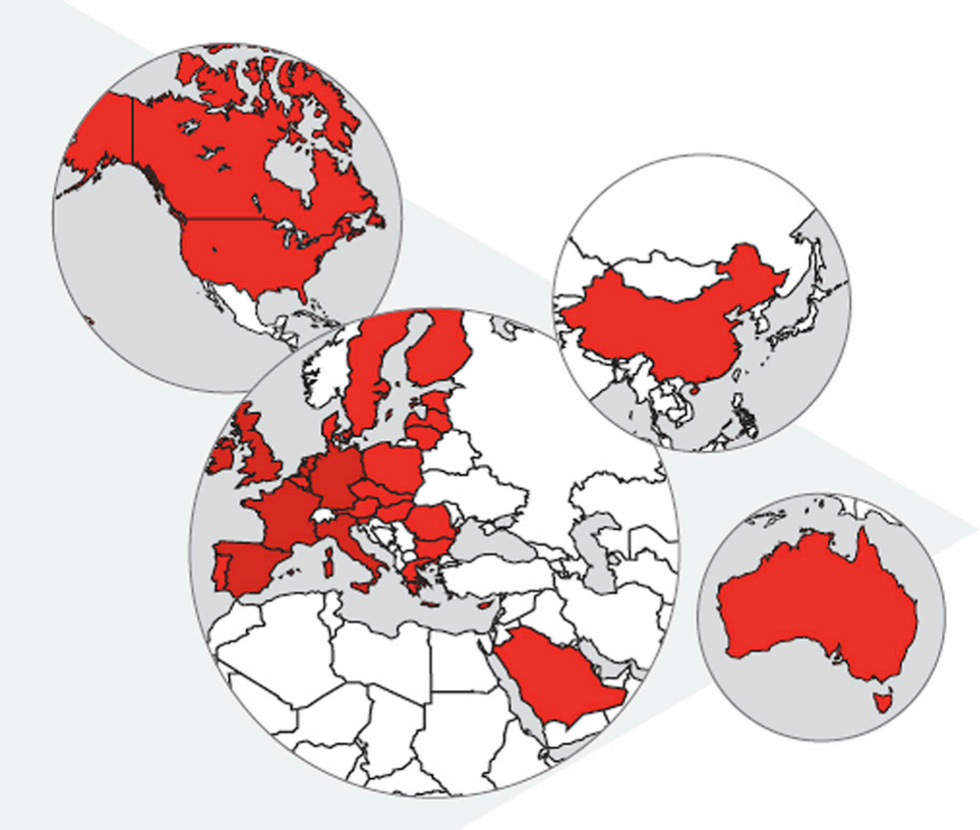
Map of the IRDiRC The IRDiRC was formally launched in 2011 and currently includes member institutions from Asia, the Middle East, Australasia, Europe, and North America. The current cumulative commitment from the 42 member institutions from both the public and private sectors is estimated at more than $2,000,000,000 USD.

The International Rare Diseases Research Consortium (IRDiRC) was established in 2011 to bring together researchers and organizations invested in rare disease research. Three IRDiRC Scientific Committees (Diagnostics, Interdisciplinary, and Therapies) and representation from three patient-advocacy groups (two from the US [National Organization for Rare Disorders (NORD) and Genetic Alliance] and one from Europe [Rare Diseases Europe-EURORDIS]), advise the Consortium Assembly (formerly the Executive Committee), which includes public research funders and private-sector members from pharma and biotech from 42 member institutions. Each has committed at least $10,000,000 USD to rare disease research within their jurisdiction (Figure 3; data accessed January 11, 2017). Currently, rare disease research coordinated under the umbrella of IRDiRC totals more than $2,000,000,000 USD. IRDiRC aims to facilitate the understanding of all rare genetic diseases.

The focus of the Diagnostics and Interdisciplinary Committees, and their associated working groups and task forces, has been identifying current and future bottlenecks to RGD discovery and suggesting strategies by which international cooperation can address them. We anticipate that several shortcomings of the present-day discovery pipeline will need to be addressed if we are to continue to make important RGD discoveries at the current pace, or even accelerate it. These include the collection and analysis of clinical and genomic data, data discovery and sharing, genetic and functional support for the establishment of disease causality, and the presence of disease mechanisms that are intractable to our current analytical and genomics-based approaches, as summarized in Table 1.

### Strategies for Enabling the Diagnosis of All RGDs

The coming years will see an expanding need for large-scale infrastructure, resources, and tools for completing the grand challenge: understanding the molecular pathogenesis of all RGDs. Over the past few years, our committees, working groups, and task forces have identified specific areas of high priority to facilitate the achievement of this goal. To this end, the IRDiRC has developed a quality indicator, “IRDiRC Recognized Resources,”^9^ on the basis of specific criteria to highlight key resources (e.g., platforms, tools, standards, and guidelines), which, if used more broadly, would accelerate the pace of discoveries.

#### Ontologies, Terminologies, and Nosologies for Exchanging Clinical Data

Understanding how genomic alterations result in different disease-related phenotypes is fundamental to human health research. In this endeavor, if careful phenotypic characterization is lacking, having genomic data, even from large numbers of individuals, is of limited value. Although we have made large strides toward enabling the sharing of genotype data, standards are not widely used for the exchange of phenotypic data. For undiagnosed RGDs, the situation is even more problematic because only a few individuals in the world might have the same undiagnosed condition. Currently, numerous ontologies, terminologies, and nosologies are used, reflecting the disparate needs and practices of different communities involved in translational research and patient care in many fields of medicine.

The IRDiRC recognizes phenotype ontologies, terminologies, and disease nosologies as critical for RGD research. The Human Phenotype Ontology (HPO)^10^, ^11^ has been recognized as a useful annotation of phenotypic abnormalities of RGDs, with the understanding that other resources might be suitable in certain situations, and is being used by RGD databases such as PhenomeCentral,^12^ DECIPHER,^13^ the UK10K Project,^14^ and many others. The HPO has been incorporated into the United Medical Language System (UMLS), which will allow interoperability with an even larger range of medical informatics resources. The HPO is more than a clinical terminology; all terms are set in a hierarchical structure, and it is designed to allow computational analysis of clinical findings for differential diagnostics,^15^ as well as RGD phenotypic stratification prior to WES analysis in both the clinical^16^ and discovery settings.^17^ A key area for ontological development is increasing the granularity and coverage of the HPO across some less well-covered rare-disease domains. Additionally, enabling a means of making longitudinal assessments (onset and temporality), utilizing phenotype negation (the patient does not have phenotype X), and making quantitative specifications (e.g., levels of abnormality of laboratory results) will be important.

To bridge the compatibility gap between various systems and the lack of terminology specific enough for RGDs, the newly established International Consortium for Human Phenotype Terminologies (ICHPT) has worked to provide the community with phenotype terminology standards and definitions for the more often used phenotype terms for database interoperability, in particular to allow the linking of phenotype and genotype databases for RGDs. The ICHPT was created with input from members of several groups, including Orphanet (under the EuroGentest project; see Web Resources), HPO,^18^ and OMIM (Robinson et al., 2014, Am. Soc. Hum. Genet., abstract). The outcome of this effort is a set of >2,300 terms that should be present in any terminology through one of its synonyms. These terms have already been mapped to a few of the major terminologies, including HPO,^11^ PhenoDB,^19^ Orphanet, Elements of Morphology,^20^ POSSUM, SNOMED, MeSH, and MedDRA, facilitating cross-compatibility between systems. Where ontologies contain more detailed terms at a finer level of granularity, these terms will map “up” to the broader aligned terms. The IRDiRC recognizes and encourages the ICHPT as the minimal set of standard terms to be used for sharing phenotypic data.

Two complementary rare-disease nosologies exist, the Orphanet Rare Disease Ontology (ORDO)^21^ and OMIM.^4^ ORDO is a structured vocabulary for rare diseases and is derived from the Orphanet database; it captures relationships between diseases, genes, and other relevant features to form a useful resource for the computational analysis of rare diseases. It integrates nosologies (classifications of rare diseases), relationships (gene-disease relations and epidemiological data), and connections with other terminologies (MeSH, UMLS, and MedDRA), databases (OMIM, UniProtKB, HGNC, Ensembl, Reactome, IUPHAR, and Geneatlas), or classifications (e.g., International Statistical Classification of Diseases and Related Health Problems-10 [ICD-10]). It should be noted that ICD-10 contains only ∼500 unique rare-disease classification codes. This deficiency is now being overcome by the development of a hierarchical rare-disease classification and coding (Orpha numbers) scheme by Orphanet, which will become the basis for inclusion of the majority of known rare diseases into ICD. Orpha numbers are now increasingly used by European healthcare systems for informatics tracing of RGDs, and their introduction is fostered by National Action Plans and Strategies for Rare Diseases and recommended by the European Commission expert group on rare diseases.^22^

OMIM has also played a central role in the naming and classification of Mendelian diseases by defining recognizable patterns of features and highlighting those that allow one condition to be distinguished from another. In general, OMIM creates separate phenotype entries on the basis of molecular etiology, that is, genetic heterogeneity. OMIM’s clinical synopsis for each phenotype includes only those features that have been reported in individuals with mutations in the disease-associated gene. Each OMIM phenotype is assigned a unique and stable identifier (MIM number) that is used in the aforementioned databases and in the biomedical literature. The IRDiRC strongly supports the continued interoperability between the rare-disease nosologies ORDO and OMIM, both of which are recognized for rare-disease classification.

#### Standards, Tools, and Resources to Facilitate Genomic Data Analyses

Our ability to analyze, annotate, and ultimately share genomic datasets is fundamental to the RGD research agenda. Currently, tools and methods for analysis and annotation are not standardized and lack interoperability; as a result, the sharing of outputs from large genomic datasets is hampered. Pipelines for analyzing DNA sequences still have much room for improvement in terms of sequence alignment, variant calling, and functional annotation and prediction, especially for more complex variation such as insertions, deletions, and the wide spectrum of structural variants,^23^ calling for a harmonized approach. This observation is supported by recent data suggesting that the limited yield of WES as reported in the literature, at least in the context of certain recessive diseases, is mostly accounted for by our limited ability to correctly call variants.^24^ An example of such a platform has been developed by the RD-Connect EU project for research and diagnosis, together with the EURenOmics and NeurOmics RGD research projects. Furthermore, existing tools will need to be made interoperable and widely adopted, and their curation and updates should be duly coordinated.

Genomic data analyses for RGD discovery are also challenged by the identification of rare variants to be prioritized for further interpretation. Investigators studying the causes of RGDs are relying heavily on WES datasets compiled by consortia, such as the Exome Aggregation Consortium (ExAC; 60,000 exomes) and the NHLBI Exome Sequencing Project (ESP; 6,500 exomes), that investigate different diseases as reference datasets for analyses, and this is proving useful in decreasing the number of variants to a manageable number for certain populations. However, many of these first comparative exome datasets have been generated from populations of Western European and North American origin. This limits pathogenic variant discovery, especially from populations that have been sparsely assessed, if sampled at all. The 1000 Genomes Project has made significant contributions to our understanding of the architecture of the human genome as a large heterogeneous population dataset. Most recently, gnomAD has aggregated 15,000 genomes and 120,000 exomes, including data from the 1000 Genomes Project and the ExAC and ESP exome datasets. Increasing such population datasets and generating and sharing datasets from populations with little to no representation in existing repositories that can be used by the RGD research community, as well as others investigating human health, will be of great importance in the future. The Global Alliance for Genomics and Health (GA4GH) is active in this space and is committed to enabling responsible and effective sharing of genomic and clinical data through a federated ecosystem approach; we support these efforts and their application to RGDs.^25^ For example, the Beacon Network, a demonstration project of GA4GH, is a global search engine for genetic variation and connects 60 databases representing every inhabited continent, enabling global discovery of genetic variation.

#### Ethical Standards to Enable Data Discovery and Sharing

The RGD research community is acutely and universally aware of the need for data discovery and sharing.^26^ Given the challenge ahead of us to understand and be able to diagnose RGDs of ever increasing rarity, the ability to share clinical and genetic data maximally has become of central importance. In this regard, the IRDiRC is collaborating with the Human Variome Project (HVP) and GA4GH to tackle major ethical, legal, and social issues and agree on standards for international data to break down existing hurdles. The IRDiRC has recognized the Framework for Responsible Sharing of Genomic and Health-Related Data^27^ as a resource on the basis of international adherence to Article 27 of the UN Declaration of Human Rights, which holds that everyone has a right “to share in scientific advancement and its benefits” and “to the protection of the moral and material interests resulting from any scientific … production of which [a person] is the author.”^28^ Recently, recommendations and models for “Data Transfer Agreements” have been published with the “IRDiRC recognized” label.^29^

The IRDIRC-HVP-GA4GH collaboration is paving the way for international recognition of common data-sharing standards. Several critical areas of data-sharing governance are currently the focus of collaborative efforts. First, the collaboration developed a “tiered” consent policy that is dependent on the context of data collection and use (clinical or research) and on the level of risk that the shared data will be identified; this policy is currently in use by the Matchmaker Exchange^30^, ^31^(MME; see below). Two related initiatives, namely the Consent Codes^32^ model and the Automatable Discovery and Access Matrix (ADA-M), seek to enable systematized representation of consent-, legal-, and institutional-based permissions and restrictions associated with research and clinical records to facilitate streamlined and appropriate discovery, sharing, and use of extant datasets. This will also help to better standardize consent-form clauses, thereby guiding best practices in both research and ethics review committees. Just as consent practices need to become interoperable so as to enable greater data sharing, so too do data-access mechanisms. Efforts are currently underway to produce a new model that would facilitate data access (registered access) and use interactions with initiatives such as MME by authorizing users through a standard online authentication and attestation process. Registered access will address different categories of potential data users (researchers, clinical care professionals, and patients), as well as different levels of data depending on their identifiability and sensitivity. Additional IRDiRC-GA4GH collaboration is underway to develop a privacy-preserving linkage system that would link data from the same individual across multiple projects while also respecting privacy. Policy for recognizing ethics review to encourage streamlined and coherent ethics review for international projects and consortia is also available. Over time, such efforts will harmonize local ethical, legal, and social policies and procedures for efficient and responsible international sharing and analysis of genomic and clinical data.

#### Genetic Evidence to Support Gene Discovery

Reports from several large-scale collaborative research initiatives, including the FORGE Canada Consortium,^33^ US Centers for Mendelian Genomics,^8^ and UK Deciphering of Developmental Disorders study,^34^ indicate that under very select circumstances (including ascertainment of multiple, thoroughly phenotyped families with the same condition), the “solve rate” for RGDs is often >50%. Reports focusing on disease-causing variants in known disease-related genes in over 9,000 cases from various clinical diagnostic settings indicate an overall success rate of ∼30%.^35^, ^36^, ^37^, ^38^, ^39^ These latter cohorts have demonstrated that a substantial fraction (25%–30%) of clinical diagnostic success depends on recent progress in the discovery of genes underlying disease. This observation in combination with the higher solve rate in the research setting suggests that the unsolved fractions of these clinical cohorts contain many discoveries.

*Case-Based Matching for Gene Discovery.* The discovery of disease-gene associations requires confirmation of pathogenic genomic variation in multiple unrelated individuals affected by the same rare disease. Our collective experience suggests that it takes approximately 2–3 years to identify an additional unrelated individual with likely pathogenic mutations in the same gene after publication of a single patient or family. Thus, a central challenge is to efficiently identify additional and unrelated persons with pathogenic variant(s) in the same gene and an overlapping phenotype. It is difficult to gauge the number of such single surviving candidate genes (containing deleterious-appearing genetic variation that remains after multiple filtering steps with segregation data and pathway and/or model-organism support from existing literature) that remain unpublished and/or in inaccessible “silos” worldwide, but we estimate it to be more than 1,000.

To address this challenge, several collaborative initiatives have developed platforms for genotype- and phenotype-driven matching algorithms^12^, ^13^, ^40^, ^41^, ^42^, ^43^, ^44^, ^45^, ^46^, ^47^, ^48^, ^49^, ^50^, ^51^, ^52^; however, a connection between these existing solutions has been lacking. Very recently, the IRDiRC Diagnostics Scientific Committee, in collaboration with each participating data-sharing service, Can-SHARE, and the GA4GH, has contributed to launching a federated platform termed the MME.^53^ This platform facilitates the identification of unsolved patients and families with similar phenotypic and genotypic profiles through a standardized application programming interface (API) and standard operating procedures.^40^ The MME enables searches of multiple databases at once, circumventing the need to separately search all services by depositing data in each one. Under this initial API, each server can treat any description arbitrarily: the level of similarity required (on either the genotype or phenotype level) before a match is triggered is left to the discretion of each service. The launch of the MME is a major step forward, and currently PhenomeCentral,^12^ GeneMatcher,^41^ DECIPHER,^13^ MyGene2,^54^ *matchbox*, and Patient Archive, representing data from more than 20,000 unrelated RGD patients, are connected to one another. However, truly optimizing this type of case-based matching and enable RGD discovery on a global scale will require improvement of international data sharing, optimization, financial support, and scaling up of such infrastructure, operating procedures, and algorithms.

#### Functional Evidence to Support Gene Discovery

*Integration of Genomic Data into Systems Biology.* Parallel to the enormous advances in gene identification through WES, other large-scale -omics approaches have been developed (e.g., proteomics, transcriptomics, and metabolomics) to aid RGD discovery and facilitate the validation of variants of unknown significance. For instance, changes in protein levels or function help to identify the disease-causing variant if more than one plausible gene has been identified through WES. Data integration across different -omics datasets on population or individual patient levels will also be required for understanding the importance of disease-modifying variants in conditions with high phenotypic variability or incomplete penetrance and for assisting the development of diagnostics and therapeutic biomarkers and will play an increasing role in developing targeted therapies. For example, RD-Connect is establishing a platform where genomic data on rare disease patients are combined with other -omics data and standardized phenotypes.^55^ Such initiatives need to be increased in number and made sustainable.

*Model Systems to Facilitate Gene Discovery.* Model-systems research (in humans, yeast, flies, worms, zebrafish, mice, and other organisms) will continue to be critical in determining the functional consequences of genomic variants in candidate disease-related genes and in discovering and validating new drug targets, candidate drugs, and other therapeutic strategies. The pace of allele discovery is outstripping our ability to understand the biological consequences of individual mutations on gene, pathway, and network function. There is an opportunity for the next generation of disease modeling to address this gap in an efficient, cost-effective, and generalizable manner with higher throughput. Improved infrastructure is required for (1) allowing clinician scientists who have discovered a disease-causing variant to be exposed to the full range of experimental tools available to them, (2) allowing experts in a variety of model organisms to apply their skills on pertinent questions of biological and clinical interest, and (3) creating efficiencies so that studies are not duplicated and existing models are utilized to their full potential. Linking clinician scientists and basic researchers early and providing seed funds for collaborative experiments would be the ultimate goals of such an effort.

One approach to accelerating collaborations between clinicians and basic researchers is to proactively identify collaborative “matches” and to provide seed funding to ignite collaborative research projects. In Canada, a national infrastructure, the “Rare Diseases: Models and Mechanisms” network, has been established to link clinicians and basic researchers as soon as disease-related genes are discovered.^56^ The network is in its second year of its 3 year funding cycle and has been successful in catalyzing collaborative links for over 40 clinician and basic-scientist matches. An alternative approach is through an “enabling” scheme, in which national funding agencies allow investigators to jointly apply for supplemental funding to existing grants. In the US, for example, administrative supplements to “R” and “P” grants are not uncommon; indeed, this model has been used by the NIH Undiagnosed Disease Program to seed research on candidate genes discovered by that effort.^57^ An integrated international virtual network allowing clinician scientists to discover relevant researchers might also be a complementary and intermediate approach.

It will also be important to stimulate the establishment and validation of novel phenotyping pipelines that have correlates in other organisms by emphasizing disease relevance, pathophysiological pathways, and high efficiency. This will accelerate the evaluation of genomic variants and candidate genes, drug and drug-target testing using disease-relevant output measures, and fundamental understanding of disease mechanisms and pathologies. Phenotyping pipelines can, in some cases, assess disease traits that resemble hallmarks of the human disorder in an obvious manner (e.g., malformations, behaviorial features, or other findings). If sufficiently specific (i.e., unique), such phenotypes can validate the relevance of a disease model. The Monarch Initiative has been working in this realm since 2009 and acts as an integrative data and analytic platform that connects phenotypes and genotypes across species. Alternatively, phenotyping pipelines can assess traits that are not linked to the disease of interest in an obvious manner but that do result from the same molecular defects underlying the disease phenotype in humans and thus represent orthologous phenotypes (“phenologs”).^58^ In addition, it will be important to develop and validate novel efficient and disease-relevant test paradigms and phenotypes that can be cross-compared between species (parallel phenotyping). Such validated disease-relevant phenotypes across organisms could provide the required output measures for overcoming current bottlenecks, such as the validation of alleles and disease-related genes, at a scale that is urgently required in the post-genome-sequence era.

### Novel Disease Mechanisms

Progress toward the discovery of the genetic basis of every RGD has been substantial over the past several years. Yet, there remain a non-trivial number of well-known rare diseases (e.g., Hallerman-Streiff syndrome, Dubowitz syndrome, VACTERL, Gomez-Lopez-Hernandez syndrome, Aicardi syndrome, and PHACE syndrome) for which, despite multiple groups’ efforts to use WES and, in some cases, WGS, the causal genetic mechanism remains elusive. The reasons that such discovery efforts fail are myriad and most likely include both technical limitations (e.g., annotation errors, missed coding and non-coding variation, and structural variation) and complex biology (e.g., extreme locus heterogeneity, tissue-specific somatic mosaicism, unusual modes of inheritance, intrafamilial allelic or locus heterogeneity, and causal synonymous variants). Approaches that overcome these barriers to RGD discovery are few in number. Moreover, the rare genetic conditions for which the genetic mechanism has yet to be identified are likely enriched with those that will not be solved easily by existing WES-based approaches. Identifying the molecular basis of conditions intractable to existing approaches requires broader and innovative application of existing discovery strategies (e.g., WGS, RNA sequencing of affected cells or tissues, and deep sequencing of tissues derived from the three major embryonic lineages); improvement of computational and statistical models for variant identification, annotation, functional prediction, and prioritization—particularly for variants in non-coding regions;^59^ and development of strategies for discovering causal genetic mechanisms. Also, temporally focused, multidisciplinary assessments that take advantage of cumulative expert clinician experience and precision phenotyping centered around single patients, such as the Undiagnosed Diseases Network International,^60^ are part of a suite of approaches to supporting the discovery of rare-disease mechanisms. The development and application of these strategies will further leverage investments that support genetic and functional approaches for the discovery of underlying genetic mechanisms.

### Critical Next Steps

Achieving the IRDiRC’s goal of a means of diagnosing all RGDs will require the discovery of the genetic mechanism underlying every disorder. This challenge—producing a complete catalog of the phenotypic characteristics of all RGDs and their corresponding causal variants, developing successful approaches to discovering the underlying etiology of RGDs caused by non-traditional modes of inheritance, and establishing tools and resources to translate this new knowledge into patient care (e.g., harmonization and adoption of international guidelines for the clinical application of NGS-based approaches)—is significant. This grand challenge can be achieved only with significant international cooperation and engagement of all relevant stakeholders at a scale the community has never seen before. Efforts to engage the research community, such as the IRDiRC and GA4GH, are of critical importance, and international coordination and funding of activities will be necessary. Improving translation and reimbursement strategies for clinical genome-wide analysis of patients with rare diseases will be essential; this is particularly important for avoiding the large number of pathogenic variants identified in known genes in research projects focused on discovery and reallocating research funding to the generation and validation of novel insights. Engaging clinical laboratories, researchers, and the patient community to share their data will be critical.

We must also recognize that as more and more genes are discovered to be associated with human disease and appropriate analytical tests are established, a significant challenge in RGD diagnosis will remain: that of interpreting a growing numbers of variants of uncertain significance. DNA diagnostics for RGD is primarily based on shared knowledge about genes, genomic variation, and phenotypes. Currently, diagnostic data are collected through a multitude of approaches by many different diagnostic laboratories and are stored in a wide variety of server systems and databases, which generally lack federated connections, i.e., “silos.” Local solutions need to be developed and implemented for storing data on genetic variants and their associated phenotypes in an easy and reproducible way with common standards and terminologies. In addition, these local systems need to be connected worldwide to form a “genetics knowledge web.” Making this type of sharing part of the normal standard of care will require community engagement. Integrating existing platforms that store clinical genetic and phenotype data (e.g., ClinVar,^61^ Leiden Open Variation Database [LOVD],^62^ and DECIPHER^13^), linking different types of data (e.g., array and sequencing), and encompassing small (single-nucleotide) to large (deletion, duplication, inversion, etc.) variants will be essential. These challenges are further compounded by the rate and impact of false-positive causative variant assignments^63^ that exist in such databases, so ultimately the curation of this knowledge by relevant experts will be the key to diagnostic precision. Variant classification as pathogenic or benign will rely heavily on the same tools that are critically needed for RGD discovery, specifically the availability of population-specific disease and control databases for a diverse range of populations, the use of orthogonal assays such as metabolomics, transcriptomics, or proteomics to clarify functional effect, and the systematic screening of mutations in disease-related genes in tractable models or cell systems. Clearly, the task of assigning pathogenicity to individual variants is mission critical to informed patient care.

Achieving a means of diagnosing all RGDs will be of great importance for patients and families. It will allow genetic counseling, better prognostication, and identification of specific health risks to the individual and will prevent unnecessary or harmful diagnostic interventions and treatments. Ultimately, such insights can be applied to genome-wide sequencing in newborns for both diagnosis and screening.^64^ In an increasing number of patients, effective drug treatment is available once the exact diagnosis (e.g., lysosomal-storage disorders or congenital myasthenic syndromes) has been established.^65^ In addition, this aim will allow more patients to participate in research cohorts for clinical trials that require a definite molecular and phenotypic diagnosis, providing potential benefit from new drugs or interventions being developed by academia and the private sector.^66^ In our view, the understanding of all RGDs will be the cornerstone of precision medicine; the power of genomics to explain these rare diseases with concomitant fundamental insights into biological processes will rapidly transform medical care for these patients and their families.

## Figures and Tables

**Table 1:** Factors Contributing to Bottlenecks in the Gene-Discovery Pipeline

Clinical data	• non-specific clinical presentations (e.g., developmental delay and hypotonia) • ultra-rare and unrecognized genetic diseases • lack of ontology encompassing the complete spectrum of human phenotypes • insufficient utilization of ontologies or 3D facial-gestalt analysis in phenotyping • inconsistent multidisciplinary approaches to patient evaluation • inability to account for and compare age-specific disease presentations
Genomic data	• technical limitations of WES (e.g., copy-number variants and structural variation are not captured well) • lack of standardized technical and informatics approaches • incompleteness of population-specific control datasets
Data discovery and sharing	• lack of a widely adopted data-sharing framework • lack of common data-sharing standards • lack of a systematic way to record data-use conditions • lack of a privacy-preserving linkage system for each research participant
Genetic evidence	• siloed datasets • lack of and use of data-sharing infrastructure
Functional evidence	• lack of standardized and moderate-throughput analyses of variant impact • lack of biological insight into the function of most human genes
Novel disease mechanisms	• lack of expertise in the analysis of non-coding variants • other mechanisms including tissue-specific mosaicism, methylation, and di- or oligogenic inheritance

## Web Resources

1000 Genomes, http://www.1000genomes.org
Can-SHARE, http://www.p3g.org/resources/can-share
ClinGen, https://www.clinicalgenome.org
ClinVar, http://www.ncbi.nlm.nih.gov/clinvar
DECIPHER, https://decipher.sanger.ac.uk
EURenOmics, http://eurenomics.eu
Exome Aggregation Consortium (ExAC) Browser, http://exac.broadinstitute.org
GeneMatcher, https://genematcher.org
Genomics England, https://www.genomicsengland.co.uk
Genotype to Mendelian Phenotype (Geno2MP), http://geno2mp.gs.washington.edu
Global Alliance for Genomics and Health (GA4GH), http://genomicsandhealth.org
gnomAD, http://gnomAD.broadinstitute.org
Human Phenotype Ontology (HPO), http://www.human-phenotype-ontology.org
Human Variome Project, http://www.humanvariomeproject.org/
International Consortium of Human Phenotype Ontologies (ICHPT), http://www.irdirc.org/ichpt
International Rare Diseases Research Consortium (IRDiRC), http://www.irdirc.org
Leiden Open Variation Database (LOVD), http://www.lovd.nl/3.0/home
Matchbox, https://seqr.broadinstitute.org
Matchmaker Exchange (MME), http://www.matchmakerexchange.org
Medical Subject Headings (MeSH), http://www.ncbi.nlm.nih.gov/mesh
Medical Dictionary for Regulatory Activities (MedDRA), https://www.meddra.org
Monarch Initiative, https://monarchinitiative.org
MyGene2, http://mygene2.org
NeurOmics, http://rd-neuromics.eu
NHLBI Exome Sequencing Project (ESP) Exome Variant Server, http://evs.gs.washington.edu/EVS
OMIM, http://omim.org
Orphanet, http://www.orpha.net
Orphanet Rare Disease Ontology, http://bioportal.bioontology.org/ontologies/ORDO
Orphanet RD-Action, http://www.rd-action.eu
Patient Archive, http://patientarchive.org
PhenoDB, https://phenodb.org
PhenomeCentral, https://www.phenomecentral.org
POSSUM, http://www.possum.net.au
Rare Diseases Models and Mechanisms Network (RDMM), http://rare-diseases-catalyst-network.ca
RD-Connect, https://platform.rd-connect.eu
SNOMED CT, http://www.ihtsdo.org/snomed-ct
UK10K, http://www.uk10k.org
